# The Potential Preventive and Therapeutic Roles of NSAIDs in Prostate Cancer

**DOI:** 10.3390/cancers15225435

**Published:** 2023-11-16

**Authors:** Hossein Maghsoudi, Farhad Sheikhnia, Przemysław Sitarek, Nooshin Hajmalek, Sepideh Hassani, Vahid Rashidi, Sadaf Khodagholi, Seyed Mostafa Mir, Faezeh Malekinejad, Fatemeh Kheradmand, Mansour Ghorbanpour, Navid Ghasemzadeh, Tomasz Kowalczyk

**Affiliations:** 1 Student Research Committee, Urmia University of Medical Sciences, Urmia 57147-83734, Iran; hoseinmaghsudy@yahoo.com (H.M.); sheikhnia.f@umsu.ac.ir (F.S.); vahidrashidi1379@gmail.com (V.R.); faez.mknj@gmail.com (F.M.); 2 Department of Clinical Biochemistry, School of Medicine, Urmia University of Medical Sciences, Urmia 57147-83734, Iran; sepideh.hassani.sh@gmail.com (S.H.); fkheradmand@yahoo.com (F.K.); navidghasemzadeh71@gmail.com (N.G.); 3 Department of Medical Biology, Medical University of Lodz, 90-151 Lodz, Poland; 4 Department of Clinical Biochemistry, School of Medicine, Babol University of Medical Sciences, Babol 47176-47754, Iran; nzn.f1995@gmail.com; 5 School of Kinesiology and Health Science, York University, Toronto, ON M3J 1P3, Canada; sadafkh@my.yorku.ca; 6 Metabolic Disorders Research Center, Department of Biochemistry and Biophysics, Gorgan Faculty of Medicine, Golestan University of Medical Sciences, Gorgan 49189-36316, Iran; cnubes@ymail.com; 7 Cellular and Molecular Research Center, Cellular and Molecular Medicine Research Institute, Urmia University of Medical Sciences, Urmia 57147-83734, Iran; 8 Solid Tumor Research Center, Cellular and Molecular Medicine Research Institute, Urmia University of Medical Sciences, Urmia 57147-83734, Iran; 9 Department of Medicinal Plants, Faculty of Agriculture and Natural Resources, Arak University, Arak 38156-88349, Iran; m-ghorbanpour@araku.ac.ir; 10 Department of Molecular Biotechnology and Genetics, Faculty of Biology and Environmental Protection, University of Lodz, 90-237 Lodz, Poland; tomasz.kowalczyk@biol.uni.lodz.pl

**Keywords:** prostate cancer, nonsteroidal anti-inflammatory drugs, NSAID, inflammation

## Abstract

**Simple Summary:**

Prostate cancer is a serious health problem for men around the world, and it is often caused by inflammation in the prostate gland. The authors of this article explore how a group of drugs called nonsteroidal anti-inflammatory drugs (NSAIDs) can help prevent and treat this disease by reducing inflammation and interfering with the growth and survival of cancer cells. We describe the various mechanisms by which NSAIDs can affect prostate cancer, such as triggering cell death, halting cell division, and cutting off blood supply. We also discuss the evidence from previous studies that support the use of NSAIDs as anti-cancer agents, either alone or in combination with other treatments. NSAIDs have a lot of promise as potential drugs for prostate cancer, but more research to understand their optimal dosage, timing, and safety is needed.

**Abstract:**

Prostate cancer (PC) is the second most common type of cancer and the leading cause of death among men worldwide. Preventing the progression of cancer after treatments such as radical prostatectomy, radiation therapy, and hormone therapy is a major concern faced by prostate cancer patients. Inflammation, which can be caused by various factors such as infections, the microbiome, obesity and a high-fat diet, is considered to be the main cause of PC. Inflammatory cells are believed to play a crucial role in tumor progression. Therefore, nonsteroidal anti-inflammatory drugs along with their effects on the treatment of inflammation-related diseases, can prevent cancer and its progression by suppressing various inflammatory pathways. Recent evidence shows that nonsteroidal anti-inflammatory drugs are effective in the prevention and treatment of prostate cancer. In this review, we discuss the different pathways through which these drugs exert their potential preventive and therapeutic effects on prostate cancer.

## 1. Introduction

Prostate cancer (PC) is the second most common cancer among men worldwide [1] and one of the leading causes of cancer-related death [2,3]. Significant risk factors for PC include advanced age, African American race, family history of PC, environmental factors, lifestyle, and chronic diseases [4]. Patients with late-stage PC, characterized by metastatic lesions and poorly differentiated cancer cells, typically have a poorer prognosis. However, patients in the early stages of the disease have a favorable prognosis if they undergo treatments such as radical prostatectomy, radiation therapy, and hormone therapy. These treatments can be concerning due to various complications [5,6,7]. Studies have identified inflammation as a primary cause of PC incidence. Both acute and chronic inflammation can result in the initiation and progression of PC [8,9,10,11]. Chronic inflammation increases carcinogenesis by promoting proliferation, angiogenesis, and metastasis, while also reducing the response to the immune system and chemotherapy agents [12]. Inflammation in PC is associated with various factors, including infection [13], the microbiome [14], obesity [15], and a high-fat diet (HFD) [16]. Given that inflammation is a major etiology of PC, it is believed that NSAIDs may not only reduce the incidence of cancer but also prevent cancer progression by suppressing various inflammatory pathways, including the induction of tumor cell apoptosis, DNA damage repair, and platelet activity suppression [17,18]. Numerous studies have investigated the relationship between reducing the risk of PC and the use of NSAIDs [19,20,21]. Furthermore, more than 30 epidemiological studies, collectively describing results on over one million individuals, have identified NSAIDs as first-line chemotherapy agents against many types of cancers [22].

**Figure 1:**
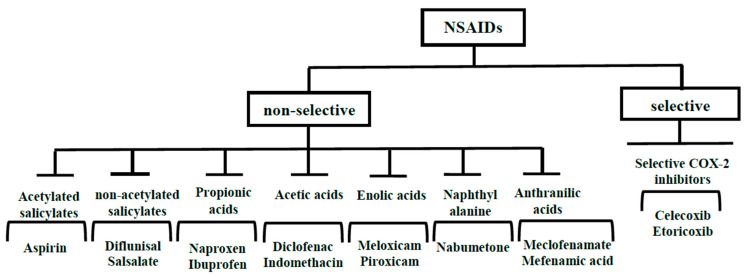
Classification of NSAIDs based on chemical structure and mechanism of action.

NSAIDs are typically categorized into several groups based on their chemical structure and selectivity: acetylated salicylates (aspirin), non-acetylated salicylates (diflunisal, salsalate), propionic acids (naproxen, ibuprofen), acetic acids (diclofenac, indomethacin), enolic acids (meloxicam, piroxicam), anthranilic acids (meclofenamate, mefenamic acid), naphthyl alanine (nabumetone), and selective COX-2 inhibitors (celecoxib, etoricoxib) [23] (Figure 1).

This review article will comprehensively discuss how some of the most commonly used NSAIDs have been employed in vitro, in vivo, and clinically in prostate cancer treatment regimens. These seven NSAIDs are frequently chosen for their potent anti-inflammatory properties. The utilization of other NSAIDs is often restricted due to their associated side effects, and there is limited research investigating their potential anti-cancer effects. In this study, our objective was to investigate the role of these commonly used NSAIDs in the context of prostate cancer.

## 2. NSAIDs

### 2.1. Aspirin

In clinical practice, aspirin (ASP) is widely used to reduce the risks of cardiovascular and cerebrovascular ischemia [24]. Numerous pharmacological, clinical, and epidemiological studies have demonstrated the protective effect of ASP against several types of cancer [25]. Cyclooxygenases (COX), the targets of NSAIDs, play a key role in homeostasis, inflammation, and immune modulation [26]. Increased expression of COX-1 and COX-2 has been observed in PC [27]. Additionally, increased production of thromboxane A2 (TXA2) and prostaglandins has been associated with the progression of PC [28]. COX-1 induces platelet aggregation and facilitates the adhesion of cancer cells to endothelial cells during metastasis [29,30]. COX-2 responds to inflammatory cytokines such as IL-1α, IL-1β, TNF-α, and lipopolysaccharide and produces increased amounts of prostaglandins in inflamed tissue. COX-2 also causes an increase in the expression of Bcl-2 in PC [18,31]. The inhibition of COX-1 and COX-2 are among the primary mechanisms through which ASP is thought to prevent cell growth [32]. ASP at low doses irreversibly inhibits COX-1 activity in platelets [33]. Inhibition of the COX-1/TXA2 pathway in platelets reduces platelet aggregation in tumor cells, endothelial activation, adhesion of tumor cells to endothelium, recruitment of metastasis-promoting monocytes/macrophages, and formation of the pre-metastatic niche in prostate tissue. High doses of ASP could inhibit COX-2 [30], resulting in the prevention of the production of prostanoids (TXA2, PGF2, PGE2, PGI2, and PGD2), which play a role in reducing apoptosis and increasing cell proliferation. It has been shown that PGE2 levels in human malignant PC tissues are 10 times higher than their levels in benign prostate tissues. PGE2 acts through receptors coupled with four G proteins named EP1, EP2, EP3, and EP4. EP3 has anti-tumor properties that can be induced by ASP. Therefore, it has been reported that ASP could exert its anti-PC properties through the activation of EP3 [34]. In another study, it was reported that ASP increased tumor necrosis factor-related ligand in PC cells by decreasing the expression of survivin, a member of the family of apoptosis-inhibiting proteins [32]. Studies have shown that ASP could inhibit the cell cycle by blocking cyclin-dependent kinases (CDKs) and causing cell cycle arrest in G0/G1 [35,36]. A retrospective case-control study demonstrated that treatment of PC cells with a combination of statin and ASP reduced both cyclin D1 expression and cell proliferation [35]. Regulatory T cells (Treg) prevent T cells from generating an effective antitumor response through immune system suppression [37,38]. A study examined the use of aspirin and statins in relation to inflammation in benign prostate tissue and revealed that aspirin could lead to a decrease in regulatory T cells [39]. COX-2/PGE2 inhibition has also been shown to reduce Treg cell activity in mouse lung cancer models [40]. In a cohort study analyzing 6594 men, ASP (low-dose < 300 mg, regular-strength 300–499 mg, extra-strength ≥ 500 mg) use was inversely associated with PC mortality [41]. Additionally, a prospective study reported that regular use of aspirin (325 mg, >3 d/wk for ≥1 yr) was associated with a reduction in the risk of lethal PC [42].

### 2.2. Ibuprofen

Ibuprofen (IBN), the most common NSAID, is typically administered to treat mild to moderate pain associated with a variety of conditions such as dysmenorrhea, headache, migraine, dental pain, and more [43]. IBN has also been reported to be effective in the prevention and treatment of certain cancers including colon, breast, lung, and gastric [44]. Several studies have been conducted to investigate the effects of IBN on PC. An in vitro study conducted in 2002 suggested that IBN had greater apoptotic and anti-proliferative effects on hormone-responsive cell lines (LNCaP and DU-145) compared to other NSAIDs including acetaminophen, ASP, naproxen, and NS-398 [45]. It was also reported that the anti-cancer effect of IBN treatment on PC3 and PC3 p53 +/+ cells was through cell cycle arrest at the G1/S stage, apoptosis induction via upregulation of caspase-3 and caspase-9, and inhibition of metastasis by upregulating E-cadherin [46].

Another study assessed the effects of R-flurbiprofen and IBN on PC-3 cancer cells and indicated that these drugs could inhibit cell migration through the induction of p75NTR, a tumor suppressor, via the p38 MAPK pathway. Furthermore, R-flurbiprofen and IBN induced the expression of the NSAID-activated gene-1 (Nag-1) protein, which was mediated by the p38 MAPK pathway. Ultimately, the inhibitory role of Nag-1 on cell migration but not survival was concluded [47]. Another mechanism of IBN’s anticancer activity on hormone-sensitive PC cell lines (PC-3 and DU-145) was mediated by blocking the constitutive activation of NF-κB and IKKα. However, NF-κB was not activated in hormone-responsive cell lines [48].

Some studies have examined IBN in combination with other compounds to investigate possible synergistic effects. For example, the combined treatment of calcitriol and IBN on LNCaP cells led to G1-S cell cycle arrest, apoptosis induction, and cell growth inhibition [49]. Moreover, the inhibitory synergistic effect of epigallocatechin-3-gallate (EGCG), a green tea component, and IBN on DU-145 PC cells was investigated. It was observed that EGCG combined with IBN could induce apoptosis through the activation of MAPK, which was shown to be blocked by N-acetyl cysteine pretreatment, demonstrating the role of oxidative stress and oxidative stress-induced activation of caspases as well as inhibition of Bfl-1 expression. This was suggested to be directly or indirectly initiated by ceramide generation [50]. In a study, the bioavailability of bulk and nanoparticle forms of ASP and IBN on lymphocytes taken from serum samples of healthy participants and PC patients was evaluated. The results showed that the nano forms of both drugs could decrease DNA damage. Micronuclei formation in lymphocytes, an index of DNA damage, decreased in nano forms and ASP bulk; however, IBN bulk led to an increase in the frequency of micronuclei in both types of samples. This provides hope that nanoparticles of NSAIDs will be effective in PC treatment [51].

The data regarding the effect of NSAID usage on PC prevention are conflicting. For instance, in a prospective study conducted in Baltimore, 141 confirmed PC cases out of 1244 participants between 1980 and 2004 were evaluated for ASP and non-aspirin NSAID usage, particularly IBN. The results showed that every use of non-aspirin NSAIDs in younger men had a significant association with a lower risk of PC diagnosis. However, in a cohort PLCO study of 29,450 participants aged 55–74 with 3575 cases of confirmed PC, the usage of ASP and IBN was evaluated. This study did not find any association between IBN and PC risk. In addition, ASP but not IBN was attributed to decreased PC risk [52]. In another case-control study carried out in Canada, the effects of five classes of NSAIDs on men aged 40 and older from 1985 to 2000 were examined. It was reported that the class of propionates including IBN and naproxen had mild effects on reducing PC risk. ASP was not observed to have protective effects in this population [53].

### 2.3. Naproxen

Naproxen (NAP) has protective effects against various cancers such as bladder, breast, and colorectal [54]. Its anti-cancerous activities are due to the upregulation of p21, p53, caspase-3, IL-10 and downregulation of PCNA, CDK4, cyclin D1, and inflammatory molecules such as iNOS, TNF-α, IL-1β, IL-4, IL-6, and IL-12 [55,56,57]. Additionally, NAP suppressed PGE2, which is a key factor in tumor progression [55]. P-glycoprotein is another target of NAP [58]. NAP exerted apoptosis by initiating the cleavage of caspase-3/7, PARP-1, and inhibiting PI3K, and Bcl-2 [57].

It is noteworthy that c-Myc and β-catenin are suppressed by NAP containing hydrogen sulfide rather than NAP and this derivative is more potent [59,60]. Several studies investigated the effects of NAP on prostate cancer. It has been shown that NAP through the induction of p75NTR, a member of the TNF receptor superfamily, increases the death of PC-3 and DU-145 [61] and LNCaP cell lines [45]. Recently, an analogue of NAP by inhibiting 17β-hydroxysteroid dehydrogenase type 5 (aldo-keto reductase 1C3/AKR1C3 which is overexpressed in PC) has shown promising effects in castration-resistant LNCaP cells [62,63]. Alongside CXB, NAP has the greatest ability to enter prostate tissue [64].

Based on clinical trials, combinational therapy of NAP (375 mg twice daily for one year) and calcitriol (45 μg once per week) was beneficial against PC and increased PSA doubling time (PSADT) in these patients [65,66]. A case-control study concluded that administration of NAP was associated with a lower risk of PC [53]. However, a 10-year cohort study found no evidence of protection by NAP consumption [67].

### 2.4. Diclofenac

Diclofenac (DCF) is widely used as an anti-inflammatory agent in degenerative joint disease and rheumatoid arthritis [68]. Additionally, DCF is a potent analgesic drug used in physical injuries and post-surgery [69]. Various in vitro and in vivo studies confirmed the anti-cancer effects of DCF in some cancer types including neuroblastoma [70], colon [71], ovarian [72], and pancreatic [73]. Regarding PC, one of the important studies about the anticancer effects of DCF was conducted by Arisan et al., reporting that DCF could elicit endothelial-mesenchymal transition in PC3 and PC3 p53 +/+ cells through ROS generation, upregulation of Snail, N-cadherin, and vimentin together with downregulation of E-cadherin without affecting p53. It could also arrest the cell cycle at G2/M, induce apoptosis through upregulating Fas, caspase-3, and caspase-9 as well as tumor suppressor genes (Bax, Bak, and Puma). DCF downregulated Bcl-x and Mcl-1 as well [46]. Additionally, DCF as a PPARγ antagonist in combination with rosiglitazone showed an additive inhibitory effect on thymidine incorporation into DNA in PC3 cells, whereas DCF antagonized the inhibitory effect of rosiglitazone on DU-145 cells [74,75].

Treatment of PC3 cells with DCF and celecoxib as COX-2 inhibitors was conducted to indicate the protumoral effect of PGE2 through EGFR-dependent positive feedback [76]. The upregulation of MYC, a well-known transcription factor involved in cell growth and cell differentiation, results in increased levels of glycolysis in cancer cells. DCF was shown to exhibit anticancer effects on the PC3 cell line by decreasing MYC expression and inhibiting glucose metabolism in tumor cells via downregulation of crucial genes such as glucose transporter-1 (GLUT1), lactate dehydrogenase A (LDHA), and monocarboxylate transporter 1 (MCT1) as a lactate transporter because higher amounts of lactate were linked to tumor progression [77]. Inoue et al. used LNCaP-COX-2 and LNCaP-Neo cell lines to study the effect of DCF on radiotherapy (RT) (0–4 Gy). DCF was found to exhibit cytotoxicity on both cell lines in a dose-dependent manner. Also, DCF treatment made LNCaP-COX-2 but not LNCaP-Neo cells sensitive to RT, though LNCaP-COX-2 cells were more resistant to radiotherapy than LNCaP-Neo cells. They also designed an in vivo study and evaluated the anti-tumor effect of topical administration of DCF on mice bearing xenograft LNCaP-COX-2 cells. A significant decrease in tumor volume in mice receiving combined DCF + RT compared to mice treated with DCF or RT alone was observed. They thus suggested DCF as a potential radiosensitizer NSAID for PC therapy [78].

### 2.5. Indomethacin

Indomethacin (IND) currently has therapeutic efficacy against severe osteoarthritis, rheumatoid arthritis, gouty arthritis, or ankylosing spondylitis [79].

AKR1C3 is an enzyme that is involved in the biosynthesis of potent androgens such as testosterone and dihydrotestosterone (DHT) and also catalyzes the conversion of PGH2 to PGF2α, which is crucial for PC cells to proliferate [80,81]. AKR1C3 has been reported to be elevated in CRPC patients and is considered as a therapeutic target in these patients [82]. It is proposed that inhibition of this bifunctional enzyme may be useful in both androgen-sensitive and androgen-insensitive conditions [83].

DU-145 overexpressing AKR1C3 cells have been shown to resist radiation therapy by enhancing the MAPK signaling pathway and inhibiting the PPARγ pathway. IND suppressed the resistance of these cells to irradiation by inhibiting AKR1C3 [80]. IND in DuCaP cells promoted apoptosis by increasing activated caspases-3 and -7 [82]. IND significantly decreased PSA mRNA and protein levels in VCaP cells [84].

In vitro and in vivo studies showed that enzalutamide-resistant CRPC cells, also known as the C4-2B MDVR cell line, upregulate proteins such as AKR1C3, HSD3B, CYP17A1, AR-V7, and c-Myc [81,85]. The fact that AKR1C3 is responsible for enzalutamide resistance was demonstrated by LNCaP overexpressing AKR1C3 cells [81] and this resistance is due to AKR1C3-induced AR-V7 overexpression [85]. Treatment of the C4-2B MDVR cell line with IND showed an upregulation in genes associated with unfolded protein response and ER stress (such as CHAC1, DDIT4, CEBPB, and ATF6), apoptosis (such as TP53, CDKN1A, and SOCS1), and downregulation in proliferative genes (such as Myc, MCM4, and CCNE2) [85]. IND exhibited its anti-cancerous effects by blocking the AR/AR-V7 signaling pathway [85].

IND alone or in combination with enzalutamide improved the xenograft PC mouse model of CWR22Rv1 cells (another enzalutamide resistant CRPC cell line) [81,85]. Immunohistochemistry assays revealed that IND significantly reduced the Ki67 index and tumor volume, mass, testosterone level, and Bcl-2. Moreover, its combination with enzalutamide had more favorable results [81,85]. In another castrated xenograft PC mouse model of VCaP cells, similar results such as reduced ERG, AR, PSA, Ki67 positive cells, testosterone, and DHT were obtained [84].

### 2.6. Mefenamic Acid

Mefenamic acid (MFA) is an NSAID that relieves dental and menstrual pain and is typically administered orally [86]. This drug has a significant protective effect against increasing lipid peroxidation, protein oxidation, TNF-α and IL-1β levels, and ultimately reduces inflammation [87,88]. In addition, it can reduce cancer cell proliferation, progression, angiogenesis, and invasion [89]. It has also been shown that MFA in combination with ionizing radiation increases apoptosis in tumor tissues and protects against DNA damage caused by ionizing radiation [90,91]. MFA is a class of NSAIDs that has high antiproliferative activity, whereas salicylates, the most common drugs used in clinical studies, show a relatively weak antiproliferative effect [92]. MFA has been reported to act as a signaler for apoptosis by inhibiting calcium uptake in cells. On the other hand, it causes apoptosis by cleaving procaspase-3 and PARP-1 [93]. MFA was found to be effective in the treatment of PC in in vitro and in vivo models [94]. As mentioned earlier, inflammation is an essential component of PC, and it has been reported that MFA reduces its biochemical progression [95]. Preclinical in vitro and in vivo studies in PC have shown that fenamic acid-derived NSAIDs such as MFA and meclofenamate have a more significant anti-neoplastic effect compared to NSAIDs previously investigated in PC. In this study, the cytotoxic effects of meclofenamic acid and MFA on human PC cell lines (LNCaP: androgen-dependent; and PC3: androgen-independent) were investigated. It was reported that clofenamic acid was a more potent antineoplastic agent than MFA [94]. In a prospective clinical trial conducted on patients with castration-resistant PC (CRPC), it was shown that the administration of MFA (1 g/day for 6 months) decreased the biochemical progress in patients with resistant PC, improved their quality of life, and increased their body mass index (BMI) [96]. Another significant effect of MFA is in patients who are treated with androgen deprivation. Although the current standard treatment for PC is androgen deprivation therapy (ADT), this treatment has side effects such as cognitive dysfunction, risk of Alzheimer’s disease, and dementia [97]. In this regard, it has been reported in a study that MFA (1 g/day for 6 months) protects against cognitive decline caused by androgen deprivation therapy in patients with PC [95].

### 2.7. Celecoxib

Celecoxib (CXB), a member of NSAIDs that specifically inhibits cyclooxygenase-2 (COX-2), has been proposed for the treatment of several neoplasms, including prostate, colorectal, breast, lung, stomach, head and neck cancers, as well as for the prevention of prostate, colorectal, breast, lung and skin cancers [98,99,100,101,102,103,104,105]. Studies have shown that CXB is effective in treating familial adenomatous polyposis, osteoarthritis, rheumatoid arthritis, primary dysmenorrhea and acute pain [106,107,108,109]. In comparison to other NSAIDs such as diclofenac, ibuprofen and naproxen, CXB has demonstrated greater absorption into the prostate in an animal study and is more suitable for chronic prostatitis/chronic pelvic pain syndrome (CP/CPPS) [64]. Additionally, CXB has shown greater cytotoxicity when compared to other cyclooxygenase inhibitors [110]. The mechanisms by which CXB can be used against these cancers are mainly due to the reduction of inflammation through COX-2 inhibition [99], other proposed mechanisms are described below:

In vitro studies have shown that CXB reduced LNCaP cell growth and NF-κB activation, as well as androgen receptor (AR) and prostate-specific antigen (PSA) expression at both mRNA and protein levels. CXB also increased apoptosis, possibly through the inhibition of ErbB receptors [110]. The Wnt/β-catenin signaling pathway can promote prostate cancer by upregulating AR expression [111], CXB has been shown to inhibit this pathway by enhancing the destruction of the TCF7L2 transcription factor [112].

Additionally, CXB induced growth inhibition, apoptosis and decreased invasiveness in PC-3, PC-3 ML and DU145 cell lines. Bcl-2, FOXM1 and MDR-1 protein levels decreased whereas activated caspases-3 and -9 increased in vitro [113,114]. However, the downregulation of Bcl-2 by CXB is controversial; there may be a relationship between CXB-induced intracellular calcium elevation and cellular death in PC-3/LNCaP cells [115,116,117,118]. In vivo studies have shown that although CXB alone can mildly reduce tumor growth, its combination with gefitinib, an EGFR inhibitor, or docetaxel, an antineoplastic drug, can significantly suppress tumor growth and Ki67 levels while increasing caspase-3 activity and apoptosis [113,114]. CXB has been shown to decrease cell growth and viability in PC-3 cells. It also induced apoptosis, reduced NF-κB, Erk1/2 and poly (ADP-ribose) polymerase-1 (PARP-1) levels and inhibited invasion or metastasis risk in these cells [116,119,120,121,122,123,124]. Similar results were obtained from LNCaP cells with or without the presence of androgen in the culture medium, in addition to the downregulation of phosphorylated Akt (pAkt) [116,125]. A combination of CXB and atorvastatin has been shown to enhance these effects [119,125,126]. CXB’s activities against cancerous cells may be p53-independent [127].

In vivo studies have indicated that combination therapy with CXB and atorvastatin can prevent the conversion of androgen-dependent (androgen-sensitive) tumors to androgen-independent (androgen-insensitive) tumors in a xenograft immunodeficient mouse model of LNCaP cells in an androgen deprivation environment [125,128]. The potential mechanisms for this action are believed to be the inhibition of IL-6 and survivin in LNCaP cells [128]. In addition to survivin, cyclin D1 has also been reported to be suppressed by CXB in other cancers such as intestinal and colon [112,129]. CXB has also been shown to reduce cell growth and invasiveness while increasing apoptosis through the modulation of PARP-1, pAkt, AR and NF-κBp65 in MDB and PDB cell lines, which are recognized as castration-resistant prostate cancer (CRPC) cells, through the reduction of p38 [123]. Combination therapy with CXB and genistein, an inhibitor of glucose transporter-1, has demonstrated greater potency against PC-3 and LNCaP cells [130,131].

CXB alone or in combination with atorvastatin has been shown to reduce tumor size and induce apoptosis [125]. Although CXB was not able to prevent prostate cancer metastasis, it was able to halt the development of metastasis in a nude mouse model of PC-3 xenograft tumors [121]. Treatment of PC-3 xenograft tumors in mice with CXB alone decreased tumor size and increased apoptosis, and its combination with atorvastatin showed greater efficacy [119,132]. In a study evaluating the effect of a high-fat diet on the progression of prostate cancer in a mouse model, treatment with CXB was shown to reduce tumor mass and affect the proliferation of tumor cells as indicated by a reduction in the ratio of Ki67 positive cells to all tumor cells. This effect was achieved through the inhibition of myeloid-derived suppressor cells and alternatively activated (or M2) macrophages by suppressing inflammatory cytokines, particularly IL-6 and IL-13, and their signaling pathways (IL-6/pSTAT3 and M2 polarization respectively) [133]. Because aging contributes to prostate cancer, CXB administration in aged mice decreased inflammation by suppressing IL-17, iNOS, TNF-α and COX-2 [134]. Although a low dose of CXB (10 mg/kg) did not change the expression of NF-κB and IL-1β serum levels [134], a higher dose (500 mg/kg) suppressed NF-κBp65 expression [135]. In transgenic adenocarcinoma of the mouse prostate (TRAMP), CXB delayed prostate tumor progression [136], decreased the number of proliferating cell nuclear antigen (PCNA) positive cells [136,137,138,139], and augmented apoptosis [137,138]. Several studies conducted on TRAMP mice have revealed various pathways through which CXB exerts its inhibitory effect on prostate cancer, including the modulation of NF-κBp65 protein levels [136,137,138,140], STAT3/pSTAT3 and IGFR1 immunoreactivity/protein levels [137], as well as AR, VEGF and Bcl-2 [138,139]. Additionally, apoptotic proteins such as p53, BAX, caspase 3, p21 and p27 were upregulated following CXB administration [138]. Furthermore, the combination of CXB with nintedanib showed strong activity against prostate tumors by reducing angiogenesis, PCNA, inflammatory CD-3+ T cells, vimentin and TGF-β [141]. Elevated levels of IGF-1 and the IGF-1/IGFBP-3 ratio are potential risk factors for prostate cancer that were reversed by CXB [142]. Low-carbohydrate and high-protein diets in combination with CXB have been shown to decrease the risk of metastasis [143]. Castration combined with CXB administration has been shown to be more beneficial in TRAMP mice [144]. In mouse models of prostate cancer, CXB demonstrated a protective effect against CRPC following androgen deprivation therapy (ADT) by suppressing specific T cells [145].

Clinical trials have shown that CXB (200 mg twice daily for 12 months or 400 mg twice daily for 6 months) can increase prostate-specific antigen doubling time (PSADT) after radical prostatectomy and/or radiation therapy [109,146,147]. Several clinical trials have been conducted and have concluded that CXB (400 mg twice daily for 6 months), when used alongside chemotherapy or radiotherapy, had no severe adverse effects on renal function and was well-tolerated [100,148]. In addition, CXB (400 mg twice daily for 4 weeks) has been shown to upregulate several tumor suppressors such as p73 and downregulate survivin [149]. However, other clinical trials (CXB; 400 mg twice daily for 4 weeks or 200 mg twice daily) did not show any significant changes in the apoptosis index, AR or PGE2 levels of prostate tumor tissue [150,151]. CXB (400 mg twice daily for 4 weeks) administration has been shown to decrease proliferation indices (MIB-1 and Ki67) and angiogenesis (VEGF, KDR and HIF-1) while increasing apoptosis [152]. Androgen deprivation therapy (ADT) combined with CXB administration may increase survival time and decrease PSA levels without producing favorable outcomes [153,154]. CXB (800 mg daily)-docetaxel combination was reported to be useful in hormone-refractory PC patients [155,156].

**Figure 2:**
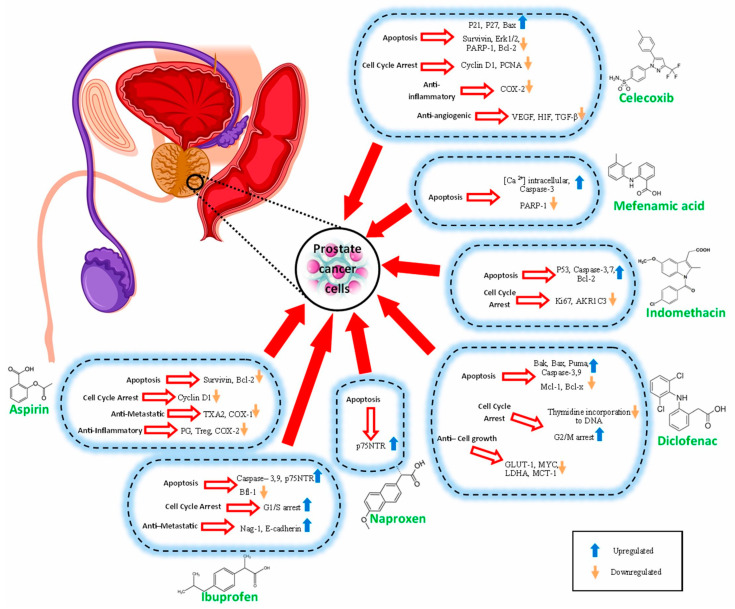
Schematic representation of the mechanisms of action of different NSAIDs in prostate cancer.

The possible anti-prostate cancer mechanisms of NSAIDs that have been discussed in various investigations are summarized in Table 1 and Figure 2.

## 3. Adverse Effects of NSAIDs

Chronic usage of NSAIDs adversely affects several organs mainly the gastrointestinal (GI), cardiovascular (CV), cerebrovascular, and renal systems; however, short-term medications and therapeutic doses could be well-tolerated [157]. The range of GI side effects differs from mild, including nausea and dyspepsia, to severe conditions like GI bleeding, perforated peptic ulcer, and iron deficiency anemia secondary to the bleeding [158]. These complications result from reduced COX-1 mucosal protective prostaglandins and decreased bicarbonate production in the stomach and small intestine. CXB is associated with minimal GI complications [159]. Among non-selective NSAIDs, DCF and NAP are the most vulnerable, whereas IBN has been reported to be safer [160].

The NSAIDs-linked renal adverse effects are acute renal failure, fluid and electrolyte (e.g., sodium and potassium) retention, GFR reduction, interstitial nephritis, papillary necrosis, and chronic renal disease [157]. Acute renal failure as the most reported NSAIDs renal complication was related to NAP and IBN [161,162]; whereas, the high dose of CXB (400 mg twice a day for six months) in a man with stage two prostate carcinoma did not have a significant effect on GFR [100]. Additionally, hypertension, atrial fibrillation, myocardial infarction, thrombotic problems caused by an imbalance between PGI2 and TXA2 production in favor of thrombosis, and heart failure have been documented [163]. Furthermore, various investigations demonstrated that cerebrovascular complications such as hemorrhagic stroke were associated with some NSAIDs in which the smallest risk was attributed to CXB and the highest to IBN, DCF, NAP, and ketoprofen [164].

To prevent the aforementioned complications, using NSAIDs at the lowest therapeutic dose for a short period is recommended [165]. Additionally, the co-administration of certain agents like proton pump inhibitors (e.g., omeprazole and pantoprazole), protective agents (e.g., misoprostol), as well as H2 receptor blockers (e.g., famotidine) will reduce the GI, especially upper GI adverse effects [166].

In the studies previously mentioned pertaining to prostate cancer, NSAIDs were generally well-tolerated. However, additional clinical trials are necessary to further ascertain the safety profile of these drugs.

## 4. Conclusions

Inflammation is strongly associated with prostate cancer and plays a key role in tumor development and progression. As such, targeting inflammation, either alone or in combination with chemotherapeutic agents, can be beneficial for the prevention and treatment of prostate cancer. A large number of studies have shown that the use of nonsteroidal anti-inflammatory drugs (NSAIDs) is associated with a decrease in cancer incidence, progression and recurrence. Additionally, NSAIDs have been shown to prevent cancer progression not only by suppressing various inflammatory pathways but also by inducing tumor cell apoptosis, protecting and repairing DNA damage, and suppressing platelet activity. Investigations have indicated that anti-inflammatory agents could be used as adjuncts to conventional cancer therapies; however, further studies are needed to better understand their potential as anticancer drugs.

## Figures and Tables

**Table 1:** Anti-prostate cancer mechanisms of NSAIDs; upregulation, downregulation.

Effects NSAIDs	Apoptosis	Cell Cycle Arrest	Anti-Metastatic	Anti-Cell Growth	Anti-Inflammatory	Anti-Angiogenic
**ASP**	Survivin, Bcl-2	Cyclin D1	TXA2, COX-1		PG, Treg, COX-2	
**IBN**	Caspase-3,9, p75NTR Bfl-1	G1/S arrest	Nag-1, E-cadherin			
**NAP**	p75NTR					
**DCF**	Bak, Bax, Puma, Caspase-3,9 Mcl-1, Bcl-x	Thymidine incorporation to DNA G2/M arrest		GLUT-1, MYC, LDHA, MCT-1		
**IND**	P53, Caspase-3,7, Bcl-2	Ki67, AKR1C3				
**MFA**	[Ca^2+^] intracellular, Caspase-3 PARP-1					
**CXB**	P21, P27, Bax. Survivin, Erk1/2, PARP-1, Bcl-2	Cyclin D1, PCNA			COX-2	VEGF, HIF-1, TGF-β
